# Chorioamnionitis induces hepatic inflammation and time-dependent changes of the enterohepatic circulation in the ovine fetus

**DOI:** 10.1038/s41598-021-89542-4

**Published:** 2021-05-14

**Authors:** Cathelijne Heymans, Marcel den Dulk, Kaatje Lenaerts, Lara R. Heij, Ilse H. de Lange, Mhamed Hadfoune, Chantal van Heugten, Boris W. Kramer, Alan H. Jobe, Masatoshi Saito, Matthew W. Kemp, Tim G. A. M. Wolfs, Wim G. van Gemert

**Affiliations:** 1 Department of Surgery, NUTRIM School of Nutrition and Translational Research in Metabolism, Maastricht University, 6200 MD Maastricht, the Netherlands; 2 Department of Surgery, Maastricht University Medical Center+, 6202 AZ Maastricht, the Netherlands; 3 Department of Surgery, University Hospital Aachen, 52074 Aachen, Germany; 4 Department of Pathology, University Hospital Aachen, 52074 Aachen, Germany; 5 Department of Pediatrics, School for Oncology and Developmental Biology (GROW), Maastricht University, 6200 MD Maastricht, the Netherlands; 6 Department of Pediatrics, Maastricht University Medical Center +, 6202 AZ Maastricht, the Netherlands; 7 Division of Obstetrics and Gynecology, The University of Western Australia, Crawley, WA 6009 Australia; 8 Division of Neonatology/Pulmonary Biology, Cincinnati Children’s Hospital Medical Center, The Perinatal Institute, University of Cincinnati, Cincinnati, OH 45229 USA; 9 Center for Perinatal and Neonatal Medicine, Tohoku University Hospital, Sendai, Miyagi 980-8574 Japan; 10 School of Veterinary and Life Sciences, Murdoch University, Perth, WA 6150 Australia; 11 Department of Biomedical Engineering (BMT), School for Cardiovascular Diseases (CARIM), Maastricht University, Universiteitssingel 50, P.O. Box 5800, 6200 MD Maastricht, the Netherlands

**Keywords:** Erythropoiesis, Infant necrotizing enterocolitis

## Abstract

Chorioamnionitis, inflammation of fetal membranes, is an important cause of preterm birth and a risk factor for the development of adverse neonatal outcomes including sepsis and intestinal pathologies. Intestinal bile acids (BAs) accumulation and hepatic cytokine production are involved in adverse intestinal outcomes. These findings triggered us to study the liver and enterohepatic circulation (EHC) following intra-amniotic (IA) lipopolysaccharide (LPS) exposure. An ovine chorioamnionitis model was used in which circulatory cytokines and outcomes of the liver and EHC of preterm lambs were longitudinally assessed following IA administration of 10 mg LPS at 5, 12 or 24h or 2, 4, 8 or 15d before preterm birth. Hepatic inflammation was observed, characterized by increased hepatic cytokine mRNA levels (5h – 2d post IA LPS exposure) and increased erythropoietic clusters (at 8 and 15 days post IA LPS exposure). Besides, 12h after IA LPS exposure, plasma BA levels were increased, whereas gene expression levels of several hepatic BA transporters were decreased. Initial EHC alterations normalized over time. Concluding, IA LPS exposure induces significant time-dependent changes in the fetal liver and EHC. These chorioamnionitis induced changes have potential postnatal consequences and the duration of IA LPS exposure might be essential herein.

## Introduction

Premature birth, before 37 weeks of gestation, is a major cause of morbidity and mortality among infants worldwide^1^. Chorioamnionitis, inflammation of the chorion and amnion during pregnancy, resulting from an ascending bacterial infection through the birth canal, is associated with premature birth^2–4^. During chorioamnionitis, the fetus can develop the fetal inflammatory response syndrome (FIRS), characterized by increased fetal systemic IL-6 and IL-8 levels^5^. Chorioamnionitis, prematurity and FIRS can contribute to significant rates of neonatal morbidity and mortality and are associated with the development of adverse neonatal outcomes including early-onset sepsis and intestinal pathologies^6–9^.

Sepsis-associated cholestasis is a common complication in infants and is commonly caused by lipopolysaccharide (LPS), derived from Gram negative bacteria^10^. This inflammation-induced cholestasis results from a reduced mRNA expression of various hepatic bile acid (BA) transporters, such as the Na^+^-taurocholate cotransporting polypeptide (*NTCP*) and the bile salt export pump (*BSEP*)^10^. In specific, LPS activates cytokine production of Kupffer cells such as tumor necrosis factor alpha (TNF-α) and interleukin 1 beta (IL-1β). These cytokines bind to their receptors on hepatocytes that activates an intracellular signaling cascade resulting in altered nuclear transcription factors and reduced mRNA expression^10^. Cholestasis can lead to hepatocyte injury as BAs have been shown to be hepatotoxic during cholestasis^11^. Several mechanisms may account for this hepatotoxity; BAs could disrupt cell membranes through their detergent action on lipid components and also promote the generation of reactive oxygen species that oxidatively modify lipids, proteins and nucleic acids, which eventually cause hepatocyte necrosis and apoptosis^11^.

The liver and intestine are closely connected via the enterohepatic circulation (EHC) of BAs. In line, BAs are recently found to be critical regulators of intestinal epithelial function^12^. Several studies have found that elevated ileal BAs and an altered expression of several BAs transporters may result in ileal damage**,** contributing to the development of necrotizing enterocolitis (NEC). More precisely, full necrosis in the ileum was found in rats as the result of intraluminal accumulation of conjugated BAs, which was similar to the histopathological findings in an experimental rat NEC model^13,14^. Increased BA synthesis might be the cause of these elevated intraluminal BAs concentrations^15^. In addition, preterm infants with NEC and rodents with induced NEC were found to have an increased expression of the apical sodium-dependent bile acid transporter (*ASBT*), a protein involved in intestinal BAs uptake, suggesting increased BAs uptake by enterocytes^14,16^. In addition, an insufficient transport from the apical to basolateral side of the enterocyte was suggested since rats with induced NEC had a decreased expression of ileal bile acid-binding protein (*IBABP*) in their ileum^14^. Altogether, this results in BAs accumulation within enterocytes, with concomitant enterocyte damage^17^, resulting from similar mechanisms as in cholestasis induced hepatocyte injury^11^. The production of hepatic cytokines was increased in neonatal rats with induced NEC and this correlated with the progression of intestinal damage during NEC development^18^, which further underlines the important role of the gut-liver axis in NEC pathogenesis.

Fecal BA levels were found to be higher in preterm infants in the week preceding NEC manifestation compared with gestation matched controls^17^. Moreover, it was shown that fetuses exposed to endotoxin-induced chorioamnionitis develop hepatic inflammation and a disturbed lipid metabolism in utero^19^, which may persist into adolescence^20^. These combined findings suggest that the mentioned earlier liver and EHC alterations might already have their origin in utero.

Recent research showed that the fetal liver is an active immune organ with the ability of inducing an early and robust innate immune response activation, and immune activation is already initiated within 1 h to 5 h after an in utero inflammatory challenge^21^. The changes during intra-uterine infection, including liver inflammation and EHC alterations, are likely to be time-dependent as inflammation is a dynamic process and the vulnerability of the fetus to injurious hits during the complex intra-uterine development varies. The aim of this study is, given the involvement of the liver in sepsis and adverse gastro-intestinal outcomes, to evaluate the time-dependent effects of intra-uterine administration of one bolus of LPS from 15 days to 5 hours before premature delivery, on the liver and EHC of premature sheep.

## Materials and methods

### Animal model and experimental procedures

This animal study was performed in Australia and the experiments were approved by the Animal Ethics Committees at The University of Western Australia (Perth, Australia; permit number: RA/3/100/928). Our study was carried out in compliance with the ARRIVE guidelines.

**Figure 1:**
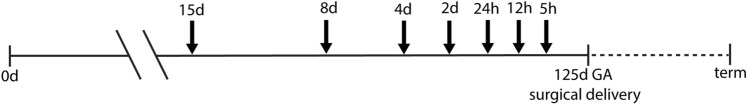
Study design. At 5, 12, or 24 hours or 2, 4, 8 or 15 days (black arrows) pregnant ewes received an IA injection with 10 mg LPS before preterm delivery at 125 days of gestation (term ~ 150 days). An IA saline injection at comparable time points to LPS injections was given to control animals. Timing shown in gestational days.

The original ovine model and experimental design were previously published^5,22–24^. In short, date-mated merino ewes pregnant with singleton fetuses were randomly assigned to eight different groups. After dropout or exclusion (fetuses in the control group with extremely high systemic IL-6 and/or IL-8 levels), the different groups consisted of 6 to 7 animals per group. The pregnant ewes received a single intra-amniotic (IA) injection under ultrasound guidance of 10 mg *Escherichia coli*-derived lipopolysaccharide (LPS) (O55:B5; Sigma-Aldrich, St. Louis, MO, USA) dissolved in saline. These injections were given at 5, 12 or 24 hours or 2, 4, 8 or 15 days before preterm delivery at 125 days of gestation (corresponds with 30–32 weeks of human gestation; term gestation in sheep is around 150 days). The control group received IA injections of saline at variable gestational ages comparable to the time points of the LPS injections before preterm delivery (Fig. 1).

The preterm lambs were delivered by cesarean section at 125 days of gestation and euthanized with intravenous pentobarbital (100 mg/kg). During necropsy, blood, liver and terminal ileum samples were sampled. Liver and ileum samples were fixed in paraformaldehyde and subsequently embedded in paraffin or liver and ileum samples were snap frozen in nitrogen.

### Qualitative analysis of liver histology

A Hematoxylin and Eosin (H&E) staining was performed which an independent pathologist blinded to the experimental set-up analyzed as previously described^25^. In short, the slides were qualitatively scored pathologically on a 0 to 4 scale for hepatic sinusoidal dilatation, shape and size of central veins and number and location of extramedullary hematopoietic clusters. Animals that had been assigned with scoring 0 had no sinusoidal dilatation, no divergent shape or size of central veins and no altered extramedullary hematopoietic clusters. Animals that had been assigned with scoring 4 had pronounced sinusoidal dilatation throughout the parenchyma, large central veins with venous stowing throughout the parenchyma and a severely increased pathologic score of extramedullary hematopoietic clusters. An increased pathologic score of extramedullary hematopoiesis, reflecting clustered and conflated hepatic erythropoiesis in the parenchyma, is a hallmark of increased erythropoiesis as a response to inflammation^26^.

### Total bile acid assay

As previously described^25^, total bile acids (tBAs) were measured in plasma, liver homogenate and terminal ileum homogenate by an enzymatic cycling method, according to the manufacture protocol (Total Bile Acids Assay kit, Diazyme Laboratories, Poway, CA, USA). tBAs in liver and ileum homogenate were corrected for protein content, which were measured with a BCA protein assay kit (Thermo Fisher Scientific, Waltham, MA, USA).

### RNA extraction and real-time PCR

RNA extraction and quantitative real-time polymerase chain reaction (qPCR) was performed as previously described^25^. In short, RNA was extracted from snap frozen liver and terminal ileal tissue using TRI reagent (Thermo Fisher Scientific)/chloroform extraction. Hereafter, with the use of a sensifast cDNA Synthesis kit (Bioline, London, UK), RNA was reverse transcribed into cDNA. With the specific primer in Sensimix SYBR & Fluorescein Kit (Bioline), a qPCR was performed using a 384-wells qPCR plate. qPCR reactions were executed in a LightCycler 480 Instrument (Roche Applied Science, Basel, Switzerland) for 45 cycles. mRNA expression levels of tumor necrosis factor alpha (*TNF-α*), interleukin 1 beta (*IL-1β*), interleukin-8 (*IL-8*) and interleukin-18 (*IL-18*) were measured to assess liver inflammation. *IL-18* mRNA expression was also measured in ileal samples to asses ileal inflammation. Gene expression levels of cholesterol 7 alpha-hydroxylase (*CYP7A1*), cytochrome P450 family 27 subfamily A member 1 (*CYP27A1*), Na^+^-taurocholate cotransporting polypeptide (*NTCP*), bile salt export pump (*BSEP*), apical sodium–dependent bile acid transporter (*ASBT*), fibroblast growth factor 19 (*FGF19*), ileal bile acid-binding protein (*IBABP*) and organic solute transporter alpha–beta (*OSTα-β*) were measured to assess alterations in the EHC of BAs. To calculate the mRNA expression levels, LinRegPCR software (version 2016.0, Heart Failure Research Center, Academic Medical Center, Amsterdam, the Netherlands) was used. The geometric mean of the expression levels of three reference genes (ribosomal protein S15 (*RPS15*), glyceraldehyde 3-phosphate dehydrogenase (*GAPDH*) and peptidylprolyl isomerase A (*PPIA*)) was calculated and used as a normalization factor. Data are shown as fold increase over the control value; arbitrary unit (AU). Primer sequences are displayed in Table 1.

**Table 1:** Primer sequences.

Primer	Forward	Reverse
RPS15	5ʹ-CGAGATGGTGGGCAGCAT-3ʹ	5ʹ-GCTTGATTTCCACCTGGTTGA-3ʹ
GAPDH	5ʹ-GGAAGCTCACTGGCATGGC-3ʹ	5ʹ-CCTGCTTCACCACCTTCTTG-3ʹ
PPIA	5ʹ-TTATAAAGGTTCCTGCTTTCACAGAA-3ʹ	5ʹ-ATGGACTTGCCACCAGTACCA-3ʹ
TNF-α	5ʹ-CATCTTCTCAAGCCTCAAATAACAA-3ʹ	5ʹ-TGCGAGTAGATGAGGTAAAGCCC-3ʹ
IL-1β	5ʹ-AGAATGAGCTGTTATTTGAGGTTGATG-3ʹ	5ʹ-GTGAGAAATCTGCAGCTGGATGT-3ʹ
IL-8	5ʹ-GTTCCAAGCTGGCTGTTGCT-3ʹ	5ʹ-GTGGAAAGGTGTGGAATGTGTTT-3ʹ
IL-18	5ʹ-AAGGGGCTGCCGTCTTCTAT-3ʹ	5ʹ-GATCTGATTCCAGGTCGCCAT-3ʹ
CYP7A1	5ʹ-GGGCATCACAAGCAAACACC-3ʹ	5ʹ-GATGATACTGTCTAGCACGGG-3ʹ
CYP27A1	5ʹ-CCCAAGAATACCCAGTTTGTGC-3ʹ	5ʹ-GGTGGCAGAAGACTCAGTTCA-3ʹ
NTCP	5ʹ-TCCTCAAATCCAAACGGCCA-3ʹ	5ʹ-GTTTGGATCGTCCATTGAGGC-3ʹ
BSEP	5ʹ-ACTCAGTAATTCTTCGCAGTGTG-3ʹ	5ʹ-ATCGAAACAATCGAAAGAAGCCA-3ʹ
ASBT	5ʹ-CATGGACCTGAGCGTCAGCAT-3ʹ	5ʹ-CACGGAGACGGGAACAACAA-3ʹ
FGF19	5ʹ-TTGATGGAGATCAGGGCGGT-3ʹ	5ʹ-CGGATCTCCTCCTCGAAAGC-3ʹ
IBABP	5ʹ-ACAAGAAGTTCAAGGTCACCG-3ʹ	5ʹ-TGATACGGCTTTATGGCCCC-3ʹ
OSTα	5ʹ-ATCCCAGGTACACGGCAGAT-3ʹ	5ʹ-ATTGAGGCCAGGACAAGCAA-3ʹ
OSTβ	5ʹ-CCGAGTAGAGGATGCAACTCC-3ʹ	5ʹ-TTTGTTTTTCCGGTGGCAGC-3ʹ

### Immunohistochemistry

Staining of ionized calcium binding adaptor molecule 1 (IBA1), a macrophage marker, in the terminal ileum was used as a marker for intestinal inflammation. For immunohistochemistry, the following antibodies were used: polyclonal rabbit anti-rat IBA1 (019-19,741; Fujifilm Wako Chemicals Europe, Neuss, Germany) and polyclonal swine anti-rabbit biotin (E0353; DakoCytomation, Glostrup, Denmark). After 4% paraformaldehyde fixation and paraffin embedding 4 µm thick sections were cut. Following rehydration, endogenous peroxidase activity was blocked by incubating sections for 20 min in 0.3% H_2_O_2_ dissolved in phosphatase buffered saline. Antigen retrieval was performed by boiling the slides in 10 mM sodium citrate buffer (pH 6.0). Thereafter, aspecific binding sites were blocked by incubating the sections with 4% normal goat serum and sections were incubated with the primary antibody overnight at 4 °C. Subsequently, sections were incubated with biotin-conjugated secondary antibody for 1 h at room temperature. IBA1 positive cells were detected with 3,3ʹ-diaminobenzidine ([DAB]; Sigma Aldrich, St Louis, MO, USA). IBA1 immunoreactivity (IBA1 positive surface area) was detected and corrected for ileal surface area with the use of Qwin Pro software (v3.4.0, Leica Microsystem, Wetzlar, Germany). IBA1 immunoreactivity is depicted as the percentage of ileal surface area positively stained for IBA1.

### Data analysis

Data are presented as median with interquartile range (IQR). Statistical analyses were carried out with GraphPad Prism (version 6.01, GraphPad Software Inc., La Jolla, CA, USA). To analyze significant differences between the groups, a nonparametric Kruskal–Wallis test followed by Dunn’s post hoc test was performed. At *P* ≤ 0.05, differences were considered statistically significant. Given the small study groups and the potential biological relevance, differences with a *P* < 0.10 are also taken into account, and described as tendencies as previously described^27^. This assumption will increase the chance of a type I error, but will decrease the chance of a type II error.

## Results

### Severe liver inflammation at day 8 and day 15 after IA LPS administration

**Figure 2:**
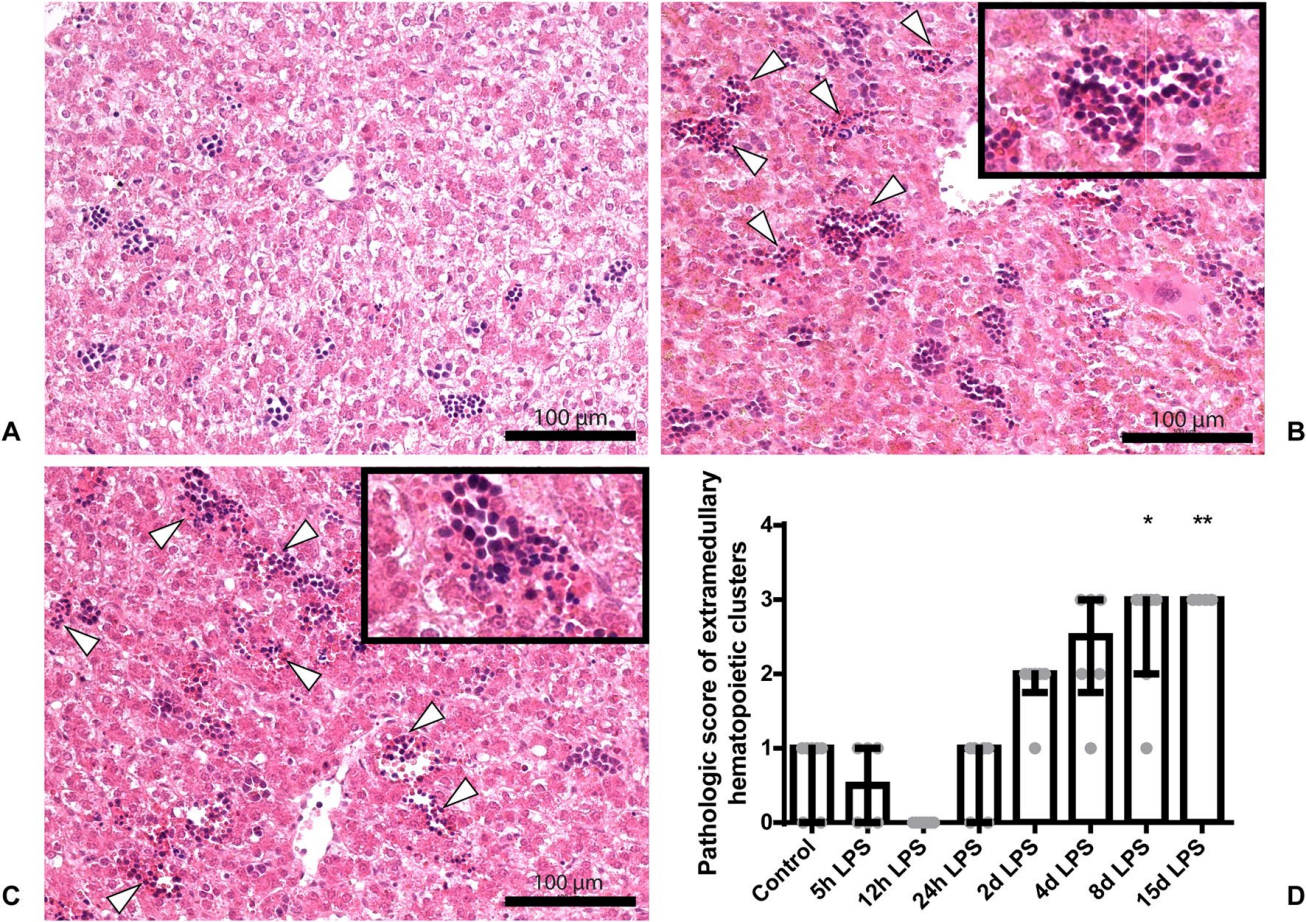
Scoring of H&E slides on a 0 to 4 scale for degree of sinusoidal dilatation, shape and size of central veins and number and location of extramedullary hematopoietic clusters. Representative images of control (**A**), at day 8 after IA LPS administration (**B**) and at day 15 after LPS administration (**C**). Data are presented as median with IQR (**D**). D: Pathologic score with increased number of extramedullary hematopoietic clusters in the animals at day 8 and day 15 after IA LPS administration. Specifically, the increased extramedullary hematopoiesis, which manifests as clustering and conflation of hepatic erythropoiesis in the parenchyma, is indicated by white triangles (b + c). *P < 0.05 and **P < 0.01 compared to control.

Histologically, no sinusoidal dilatation or divergent shape or size of central veins was observed in any of the groups. An increased qualitative pathological score of extramedullary hematopoiesis was observed in the liver of animals 8 days and 15 days after LPS administration, compared to control (both *P* < 0.05; Fig. 2A–D) reflecting clustered and conflated hepatic erythropoiesis in the parenchyma (Fig. 2B,C).

**Figure 3:**
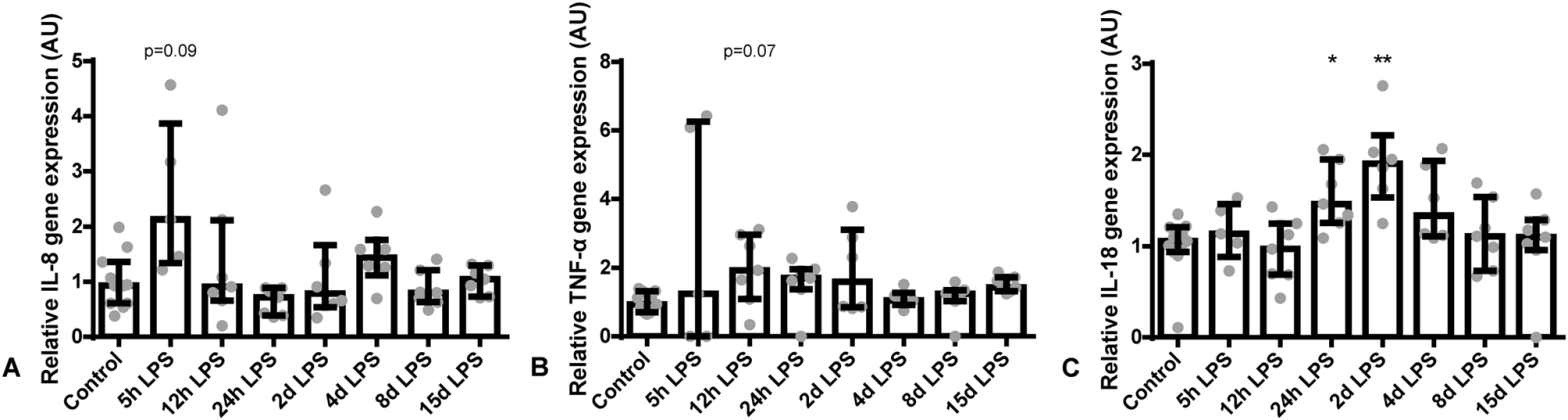
Relative mRNA expression of *IL-8* (**A**), *TNF-α* (**B**) and *IL-18* (**C**) in AU in the liver. Data are presented as median with IQR. A: *IL-8* tended to increase 5 hours after IA LPS administration. *P* = 0.09 compared to control. B: *TNF-α* mRNA levels tended to be increased 12 hours after IA LPS administration. *P* = 0.07 compared to control. C: *IL-18* mRNA levels were increased at 24 hours and 2 days after IA LPS administration. *P = 0.05 and **P < 0.005 compared to control.

Hepatic *IL-8* mRNA levels tended to be increased 5 hours after IA LPS administration, compared to control (*P* = 0.09; Fig. 3A). Furthermore, *TNF-α* mRNA levels tended to be increased 12 hours after IA LPS, compared to control (*P* = 0.07; Fig. 3B). Moreover, *IL-18* mRNA levels were increased 24 hours and 2 days after IA LPS administration (both *P* ≤ 0.05; Fig. 3C). Hepatic *IL-1β* mRNA levels did not differ between the groups (Supplementary Figure S1).

### Enterohepatic circulation alterations due to IA LPS exposure

**Figure 4:**
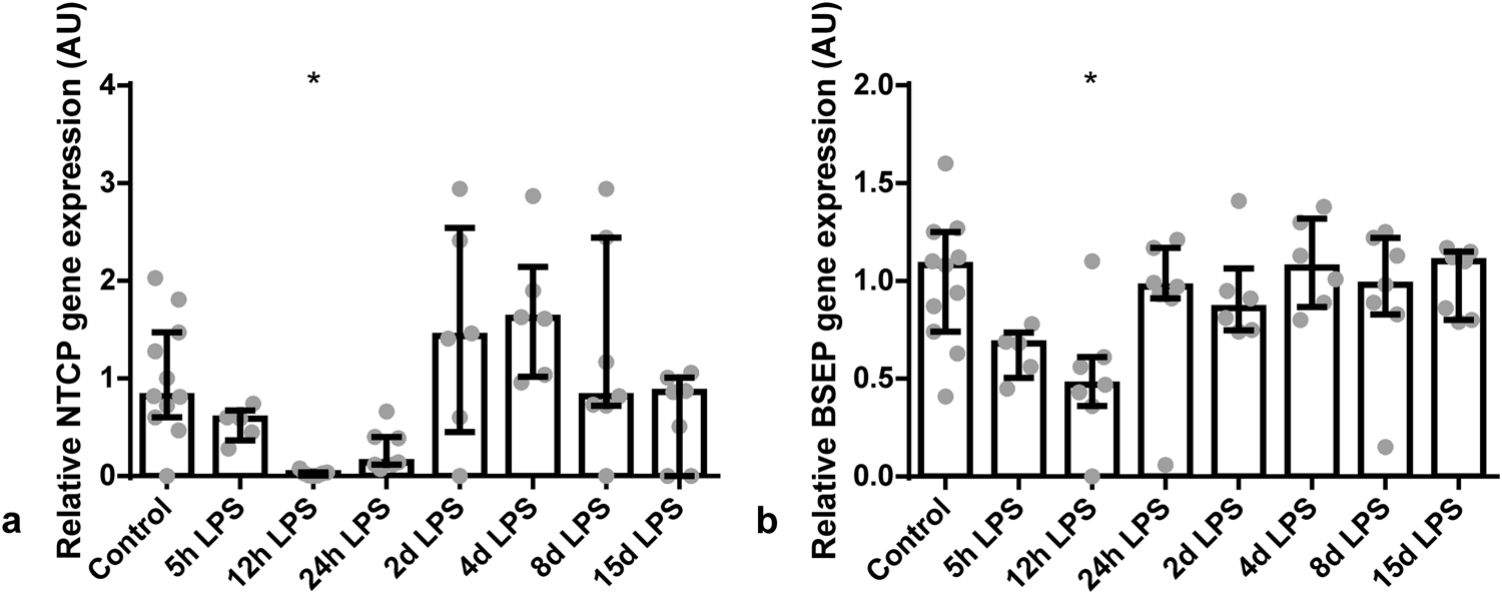
Relative hepatic *NTCP* and *BSEP* gene expression in AU. Data are presented as median with IQR. *NTCP* (**a**) and *BSEP* (**b**) were decreased 12 hours after IA LPS administration. *P < 0.05 compared to control.

Gene expression of *NTCP*, the transporter responsible for BAs uptake from the portal circulation into the hepatocyte, was decreased in the liver of animals at 12 hours after IA LPS administration, compared to control (*P* < 0.05; Fig. 4a). In addition, gene expression of *BSEP*, the pump responsible for BAs excretion from the hepatocyte into the bile canaliculi for export into the gastrointestinal tract, was also decreased in the liver of animals at 12 hours after IA LPS administration (*P* < 0.05; Fig. 4b).

**Figure 5:**
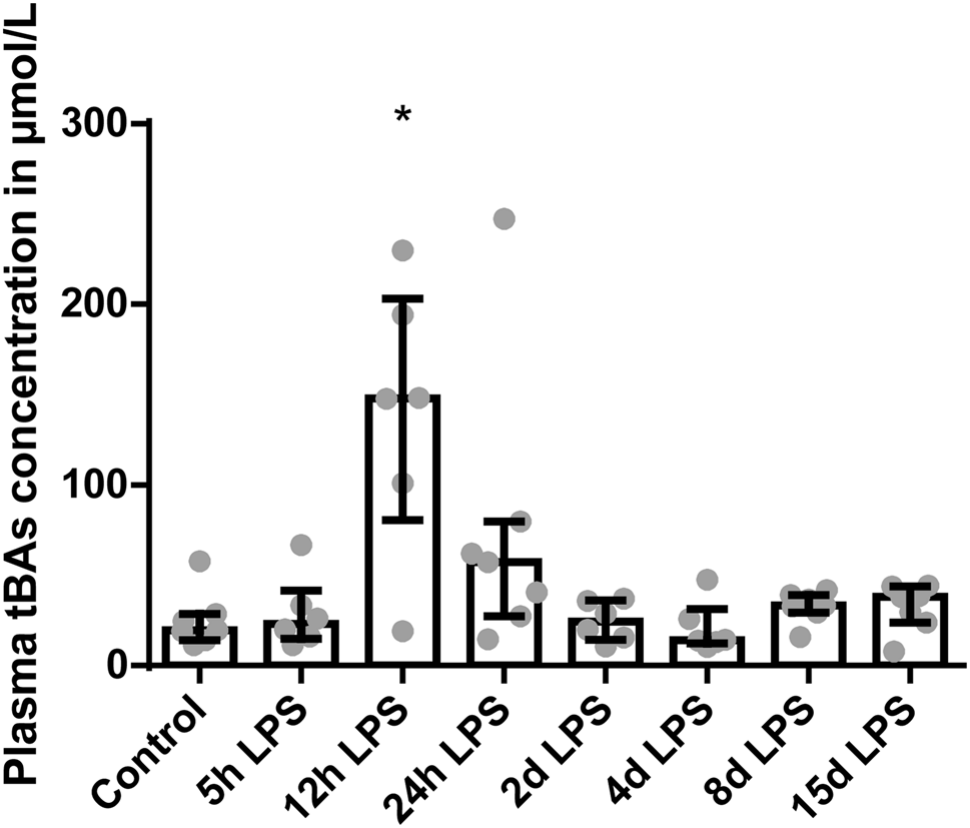
tBAs concentrations in plasma in μmol/L. Data are presented as median with IQR. Increased tBAs concentration in animals 12 hours after IA LPS administration. *P < 0.05 compared to control.

In addition, plasma tBAs concentrations were increased in animals 12 hours after IA LPS administration, compared to control (*P* < 0.05; Fig. 5). Furthermore, tBAs concentrations in liver and ileum homogenates did not differ between the groups (Supplementary Figure S2). Gene expression levels of *CYP7A1* and *CYP27A1*, BA synthesis markers, did not differ among the groups (Supplementary Figure S3).

**Figure 6:**
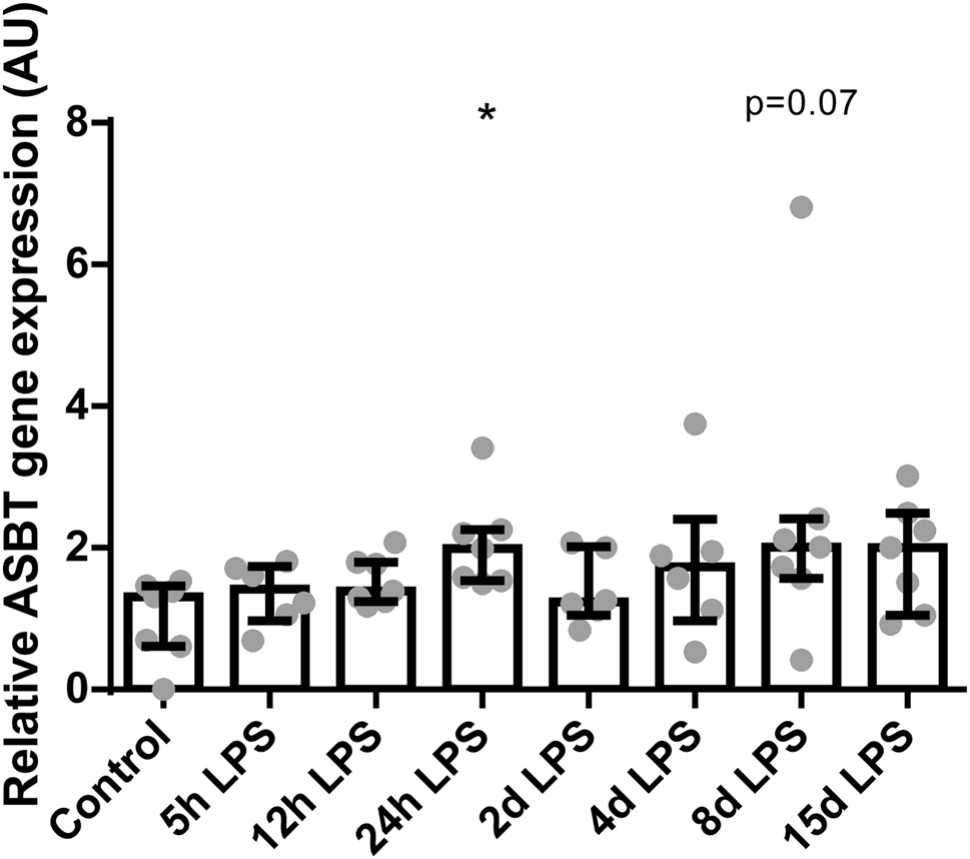
Relative gene expression of *ASBT* in AU in the terminal ileum. Data are presented as median with IQR. Increased *ASBT* expression in animals 24 hours and 8 days after IA LPS administration. *P < 0.05 compared to control. *P* = 0.07 compared to control.

**Figure 7:**
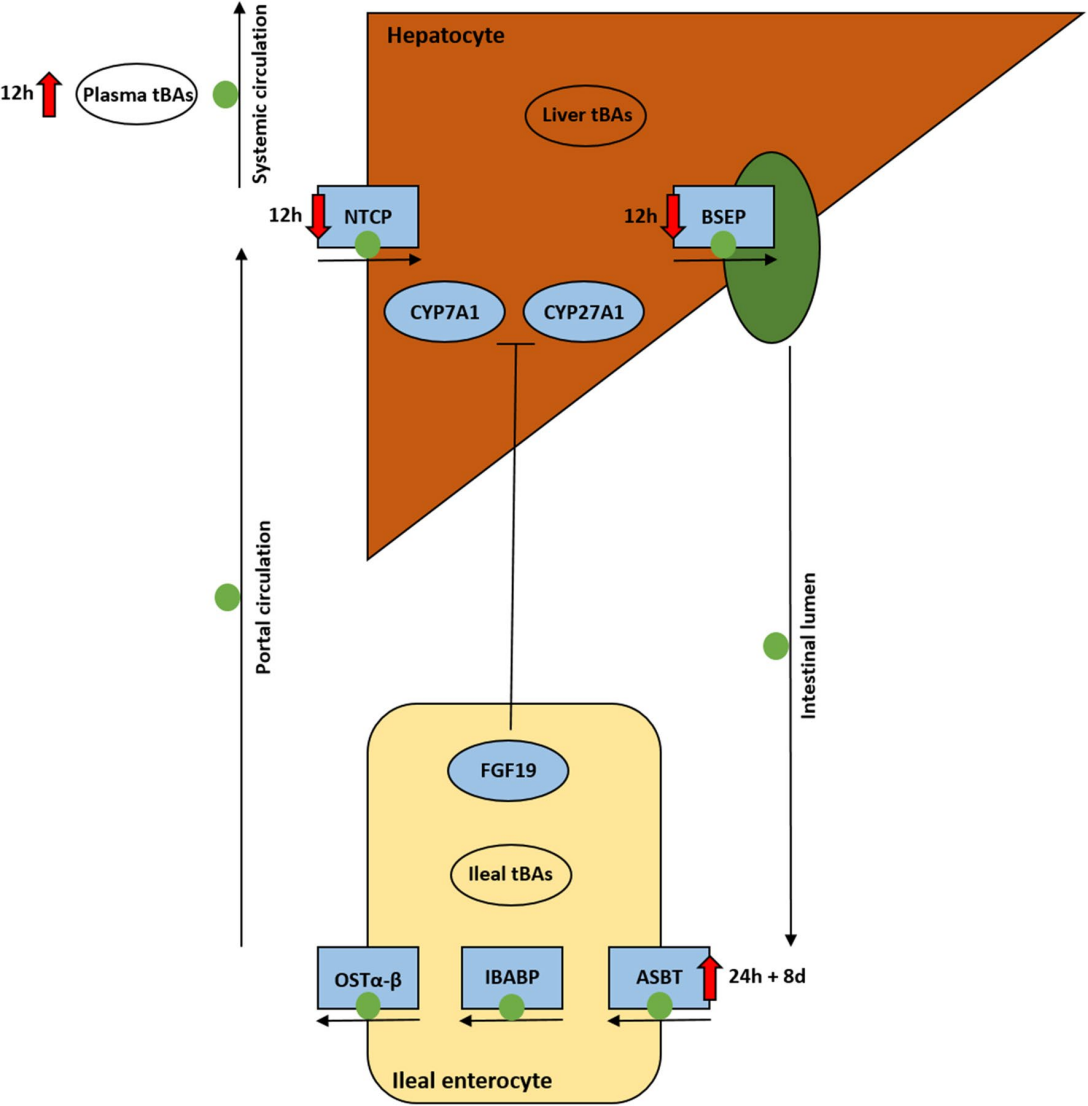
An overview of the enterohepatic circulation (EHC) of bile acids (BAs) and changes herein related to FIRS and liver inflammation. *CYP7A1* and *CYP27A1* play a role in BAs production, which can be inhibited by *FGF19*. Physiologically, BAs are transported from hepatocytes via *BSEP*, via the gallbladder subsequently into the intestinal lumen. Most BAs are reabsorbed in the terminal ileum by *ASBT*. Following enterocyte uptake, BAs bind to *IBABP* to traverse the cytoplasm of epithelial cells. BAs exit the basolateral site of the enterocyte via *OSTα*-*OSTβ*. Via the portal vein, BAs are transported back to the liver. *NTCP* takes up the BAs into the hepatocyte to be recycled. Decreased expression of *NTCP* and *BSEP* and resultant increased plasma BA levels are associated with FIRS and liver inflammation. Increased *ASBT* expression may also be inflammation dependent, or a compensatory mechanism to the postulated lower intraluminal BAs supply to maintain a constant BA pool in the EHC.

Intestinal gene expression of *ASBT*, a transporter responsible for the uptake of BAs from the lumen into the enterocyte, was increased in animals 24 hours after LPS administration (*P* < 0.05; Fig. 6) and tended to be increased 8 days after IA LPS administration, compared to control (*P* = 0.07; Fig. 6). Furthermore, intestinal mRNA expression levels of *FGF19* (a hormone regulating bile acid synthesis), *IBABP* (required for efficient apical to basolateral transport of conjugated BAs in ileal enterocytes) and *OSTα-β* (transporters responsible for BAs excretion from the enterocyte into the portal circulation) did not differ between the groups (Supplementary Figure S4). An overview of the EHC changes is depicted in Fig. 7.

### Intestinal inflammation after IA LPS exposure

**Figure 8:**
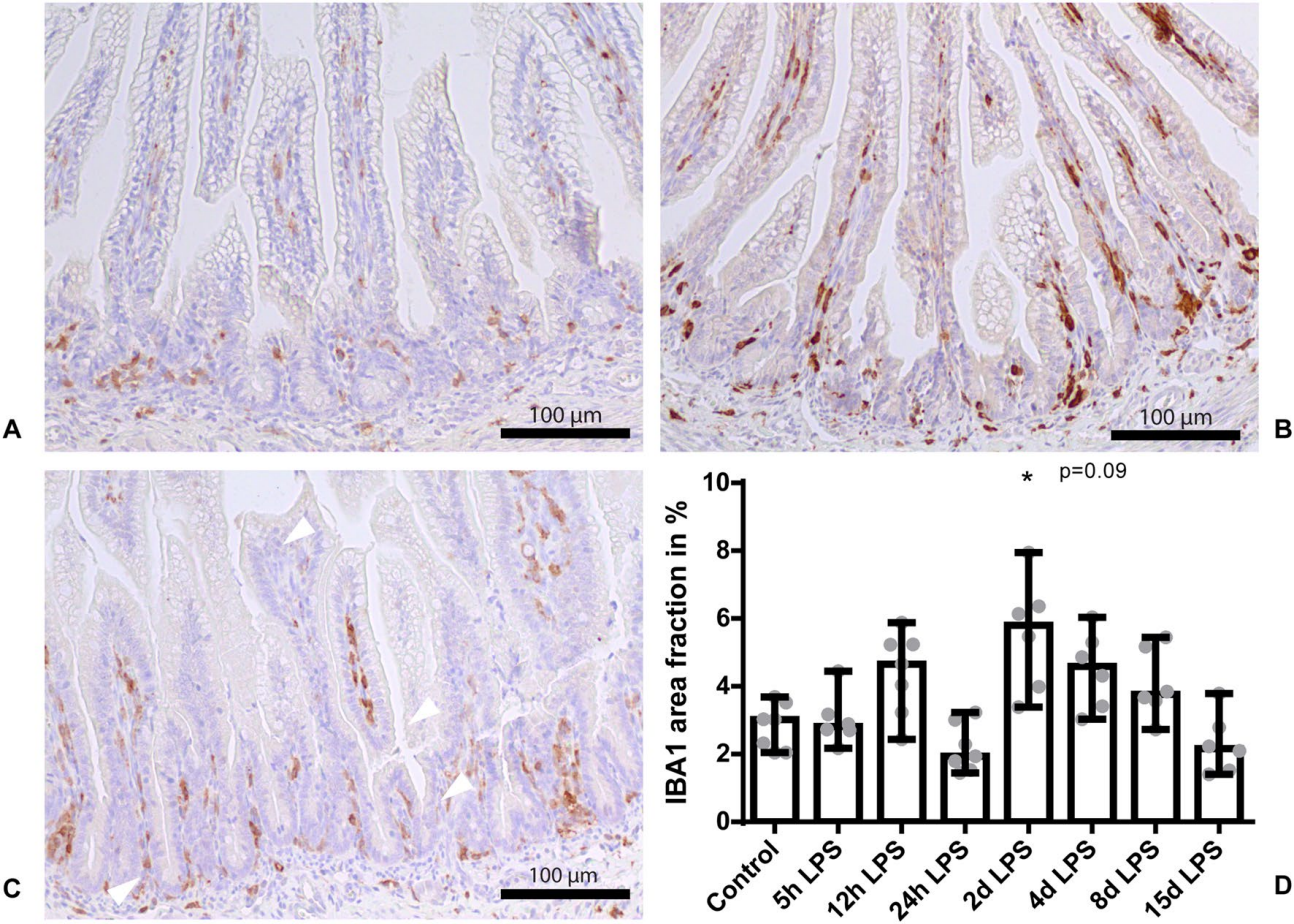
IBA1 immunoreactivity in the terminal ileum. Representative images of control (**A**), at day 2 after IA LPS administration (**B**) and at day 4 after LPS administration (**C**). Data are presented as medium with IQR (**D**). D: Increased IBA1 positive surface area in animals 2 days and 4 days after IA LPS exposure. *P < 0.05 compared to control. *P* = 0.09 compared to control.

**Figure 9:**
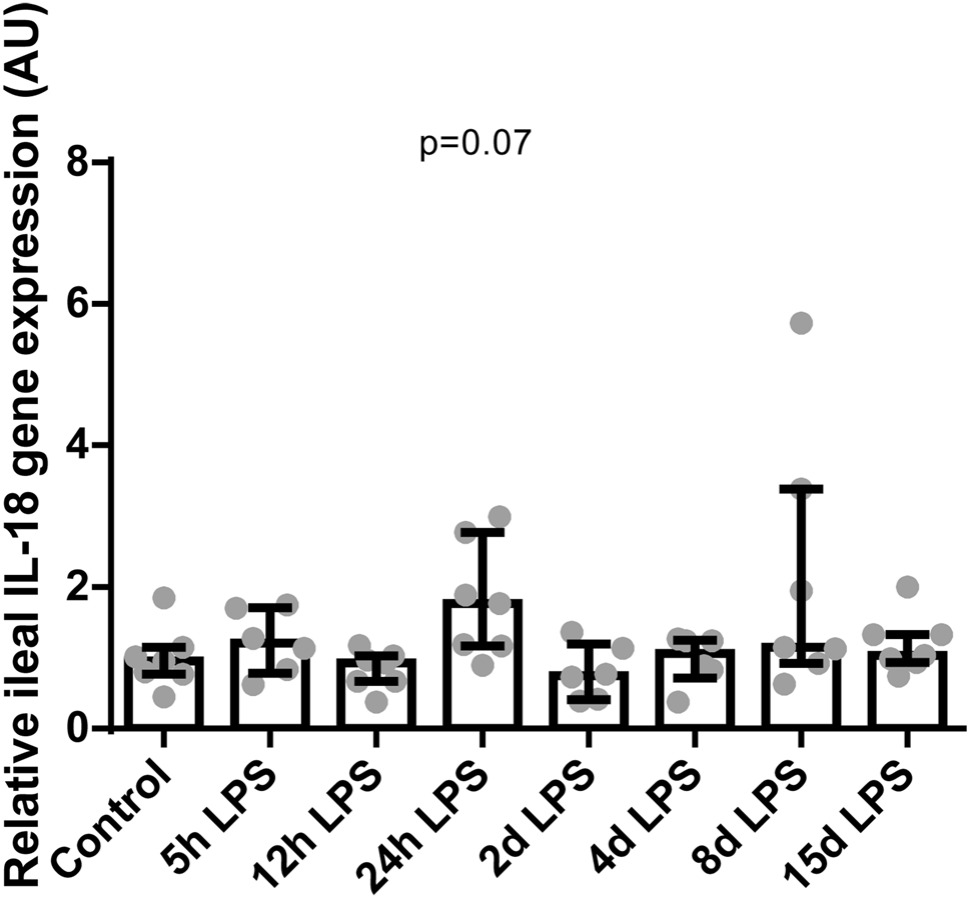
Relative gene expression of *IL-18* in AU in the terminal ileum. Data are presented as median with IQR. *IL-18* mRNA levels tended to be increased 24 hours after IA LPS administration. *P* = 0.07 compared to control.

Intestinal inflammation was detected by increased immunoreactivity of IBA1 (macrophages) in the terminal ileum of preterm lambs 2 days (*P* < 0.05; Fig. 8B,D) and 4 days (*P* = 0.09; Fig. 8C,D) after IA LPS administration, compared to control (Fig. 8A,D), which supports and extends earlier findings^24,28^. Moreover, ileal *IL-18* mRNA levels tended to be increased 24 hours after IA LPS administration, compared to control (*P* = 0.07; Fig. 9).

### Ethical approval

All procedures performed in studies involving animals were in accordance with the ethical standards of the institution at which the studies were conducted and ethical approval was obtained from the Animal Ethics Committees at The University of Western Australia (Perth, Australia; permit number: RA/3/100/928). All institutional and national guidelines for the care and use of laboratory animals were followed.

## Discussion

In this study, we evaluated the time-dependent effects of intra-uterine administration of LPS from 15 days to 5 hours before premature delivery, on the liver and EHC of premature sheep.

Recent research showed that the fetal liver is an active immune organ with the ability of inducing an early and robust innate immune response activation, and immune activation is already initiated within 1 hour to 5 hours after an in utero inflammatory challenge^21^. In our study, hepatic cytokine mRNA expression was increased from 5 hours until 2 days after IA LPS administration and these time points overlap with the ongoing fetal systemic immune response, characterized by increased circulatory *IL-6* levels^5^, suggesting that FIRS and local cytokine production in the liver are associated with each other. In accordance with these findings, chorioamnionitis-induced hepatic inflammation was associated with FIRS in a similar ovine model in which fetuses were assessed at day 2 and day 14 after IA LPS administration^19^. Accordingly, in utero, the liver might thus be one of the first organs to induce an innate immune response by early cytokine production as a result of chorioamnionitis.

An increased qualitative pathologic score of extramedullary hematopoiesis was observed in the liver parenchyma at 8 and 15 days after IA LPS administration. This increased score, reflecting clustered and conflated hepatic erythropoiesis in the parenchyma, is a hallmark of increased erythropoiesis in response to inflammation^26^. Interestingly, in human stillborns, a similar histological pattern was strongly associated with chorioamnionitis^29^, suggesting that antenatal inflammation alters fetal extramedullary hematopoiesis. Although hepatic *TNF-α*, *IL-8* and *IL-18* levels have all been normalized at day 4 after IA LPS administration and no alterations in extramedullary hematopoiesis were observed until day 8 after IA LPS administration, it is possible that the early hepatic cytokine response contributed to the altered fetal erythropoiesis at day 8 and day 15 after IA LPS administration. In addition, fetal ileal inflammation was observed in the current and previous studies^24^ with the most evident signs of inflammation 2 days and 4 days after IA LPS exposure. Transport of inflammatory mediators from the gut to the liver via the portal vein may therefore also contribute to hepatic inflammation, illustrated by extramedullary hematopoiesis. As seen in a previous study, it has been shown that upon infection and resultant immune responses, various hematopoietic factors including TLR ligands and cytokines promote extramedullary hematopoiesis in the liver^30^. Therefore, the ongoing fetal systemic inflammatory response^5^ or the direct exposure to inflammatory mediators through the transport from the gut via the portal vein, or a combination of both are likely the cause of the observed altered fetal extramedullary hematopoiesis in our study. Interestingly, increased production of hepatic cytokines was also found in neonatal rats with NEC and correlated with the progression of intestinal damage during disease development^18^. Since increased hepatic *TNF-α*, *IL-8* and *IL-18* levels preceded and showed overlap with intestinal inflammation, it is likely that the early hepatic cytokine response contributed to intestinal inflammation. Whether hepatic cytokines contribute to intestinal inflammation in utero via the periphery or the bile remains to be elucidated^18^.

The important role of the liver and the gut-liver axis in NEC pathogenesis is further emphasized by the fact that elevated ileal BAs and an altered expression of several BAs transporters can contribute to the development of NEC^13–17,31^. In our study, however, ileal BA levels were not changed. Only at 12 hours after IA LPS administration, plasma BA levels were increased, probably as a result of decreased *NTCP* expression. This indicates overflow of BAs returning via the portal circulation into the systemic circulation, with a rise in systemic BA levels as a result. Interestingly, cytokines are found to be key mediators in regulating hepatic expression of BA transporters during inflammation^32^. Specifically, IL-6 can suppress *NTCP* and *BSEP* expression levels^10,32^. The decreased expression of *NTCP* and *BSEP* in our study was thus most likely the result of the increased systemic IL-6 levels^5^. Moreover, it was shown that LPS activates cytokine production of Kupffer cells, such as TNF-α, which in turn bind to their receptors on hepatocytes resulting in reduced mRNA expression of several BAs transporters^10^. Therefore, the increased hepatic *TNF-α* expression, in our study, might also have contributed to the decreased mRNA expression of *NTCP* and *BSEP*^10,15,33^. This suggests that both FIRS and liver inflammation are causally related to the changes in the hepatic BA transporters.

Reduced mRNA expression of various hepatic excretory BA transporters, e.g. *BSEP*, was observed in many animal models upon LPS treatment and also in patients with inflammation-induced cholestasis^10^. The decreased *NTCP* expression might thus be a compensatory mechanism to protect the liver against damage during sepsis as BAs have been shown to be hepatotoxic during cholestasis^11^. Our results probably show an earlier disease state in which LPS induced sepsis not yet has progressed to cholestasis since hepatic BA levels were unchanged, indicating that these animals were not cholestatic. Another compensatory mechanism might be the unchanged BAs synthesis, which in the normal situation would be expected to increase to remain a constant BA pool.

Intestinal *ASBT* mRNA expression was increased in animals 24 hours after IA LPS administration, which might be a response to the decreased transport of BAs from the liver to the gut 12 hours after IA LPS administration. No changes in BAs concentrations in the ileum were observed at any of the time points, suggesting that the increased *ASBT* expression may be a compensatory mechanism to the postulated lower intraluminal BAs supply to keep a constant BA pool in the EHC, as *ASBT* is known to play a major role in BA homeostasis^34^. Moreover, it might also be related to the increased cytokines such as ileal *TNF-α* and *IL-18* levels, which in previous studies have been shown to upregulate *ASBT* expression^35,36^. Of note, intestinal BAs did not contribute to intestinal inflammation^24^, as they were not increased.

The elevated BA levels in serum following IA LPS exposure were also measured in neonates with NEC in combination with depressed biliary BA levels, suggesting a failure of BA transport from the hepatocytes into the bile canaliculi^37^. In addition, increased *ASBT* expression has been found in preterm infants and rodents with NEC^14,16^. However, in our study, initial alterations to the EHC normalized after IA LPS administration, suggesting that the duration of IA inflammation is important for the hepatic outcome. There is a defined window of vulnerability in which additional inflammatory hits, a premature born child is likely to encounter, might induce (additional) injury. This study highlights that the liver must be considered in neonatal care of preterm infants that suffered from inflammatory stress. Importantly, additional inflammation may have further impact on the liver and EHC and as a consequence the host in general. Based on our current findings, immune modulatory interventions such as nutrition and cytokine inhibitors might have the potential to improve neonatal wellbeing. In this context, timing of treatment initiation on the liver and EHC should be further studied.

A limitation of this study is the set-up in which the fixed moments of premature birth cannot exclude a potential influence of gestational age at start of IA infection. Furthermore, an unavoidable shortcoming of large animal studies is the relatively low number of animals per group.

In summary, in utero, the liver might be one of the first organs to induce an innate immune response by early cytokine production as a result of chorioamnionitis. An altered fetal erythropoiesis as a reaction to inflammation was detected at day 8 and day 15 after IA LPS administration that might be the result of hepatic cytokine production, ileal inflammation and ongoing FIRS. This ongoing fetal systemic inflammation and liver inflammation most likely also caused the changes in the decreased expression of several hepatic BAs transporters and resultant increased plasma BA levels that were observed 12 hours after IA LPS administration. Initial alterations to the EHC normalized over time, suggesting that the duration of IA inflammation is important for the hepatic outcome. Since a premature born child is likely to encounter additional postnatal inflammatory hits that might induce (additional) injury, this study highlights that the liver must be considered in neonatal care of preterm infants that suffered from inflammatory stress.

## Supplementary Information

Supplementary Figures.

## Data Availability

The datasets generated during and/or analysed during the current study are available from the corresponding author on reasonable request.

## Abbreviations

ASBT: Apical sodium–dependent bile acid transporter
AU: Arbitrary unit
BAs: Bile acids
BSEP: Bile salt export pump
CYP7A1: Cholesterol 7 alpha-hydroxylase
CYP27A1: Cytochrome P450 Family 27 Subfamily A Member 1
EHC: Enterohepatic circulation
FGF19: Fibroblast growth factor 19
FIRS: Fetal inflammatory response syndrome
GAPDH: Glyceraldehyde 3-phosphate dehydrogenase
IA: Intra-amniotic
IBA1: Ionized calcium binding adaptor molecule 1
IBABP: Ileal bile acid-binding protein
IL: Interleukin
IQR: Interquartile range
LPS: Lipopolysaccharide
NEC: Necrotizing enterocolitis
NTCP: Na + -taurocholate cotransporting polypeptide
OSTα: Organic solute transporter alpha
OSTβ: Organic solute transporter bèta
PPIA: Peptidylprolyl isomerase A
qPCR: Quantitative real-time PCR
RPS15: Ribosomal protein S15
tBAs: Total bile acids
TNF-α: Tumor necrosis factor alpha
