# Supplementary material for: Chorioamnionitis induces hepatic inflammation and time-dependent changes of the enterohepatic circulation in the ovine fetus
Source: Sci Rep. 2021 May 14;11:10331. doi: 10.1038/s41598-021-89542-4 (PMC8121927; doi:10.1038/s41598-021-89542-4)
Supplement: Supplementary file 1 — Supplementary Figures. [file 41598_2021_89542_MOESM1_ESM.docx]

**Chorioamnionitis induces hepatic inflammation and time-dependent changes of the enterohepatic circulation in the ovine fetus**

Cathelijne Heymans MD^1^, Marcel den Dulk MD,PhD^2,3^, Kaatje Lenaerts PhD^1^, Lara R. Heij MD, PhD^1,3,4^, Ilse H. de Lange MD^1,5^ ,Mhamed Hadfoune^1^, Chantal van Heugten^1^, Boris W. Kramer MD, PhD^5,6^, Alan H. Jobe MD, PhD^7,8^, Masatoshi Saito MD, PhD^7,9^, Matthew W. Kemp PhD^7,10^, Tim G.A.M. Wolfs PhD^5,11*^, Wim G. van Gemert MD, PhD^1,2,3^

^1^ Department of Surgery, NUTRIM School of Nutrition and Translational Research in Metabolism, Maastricht University, 6200 MD Maastricht, the Netherlands; [cathelijneheymans@hotmail.com](mailto:cathelijneheymans@hotmail.com) (CH), [kaatje.lenaerts@maastrichtuniversity.nl](mailto:kaatje.lenaerts@maastrichtuniversity.nl) (KL), [l.heij@maastrichtuniversity.nl](mailto:l.heij@maastrichtuniversity.nl) (LH), [i.delange@maastrichtuniversity.nl](mailto:i.delange@maastrichtuniversity.nl) (IL), [m.hadfoune@maastrichtuniversity.nl](mailto:m.hadfoune@maastrichtuniversity.nl) (MH), [chantalvanheugten@hotmail.com](mailto:chantalvanheugten@hotmail.com) (CvH), [wim.van.gemert@mumc.nl](mailto:wim.van.gemert@mumc.nl) (WG); +31 43 388 2222

^2^ Department of Surgery, Maastricht University Medical Center +, 6202 AZ Maastricht, the Netherlands; [marcel.den.dulk@mumc.nl](mailto:marcel.den.dulk@mumc.nl) (MD); +31 43 387 6543

^3^ Department of Surgery, University Hospital Aachen, 52074 Aachen, Germany; +49 241 800

^4^ Department of Pathology, University Hospital Aachen, 52074 Aachen, Germany; +49 241 800

^5^ Department of Pediatrics, School for Oncology and Developmental Biology (GROW), Maastricht University, 6200 MD Maastricht, the Netherlands; [b.kramer@maastrichtuniversity.nl](mailto:b.kramer@maastrichtuniversity.nl) (BK), [tim.wolfs@maastrichtuniversity.nl](mailto:tim.wolfs@maastrichtuniversity.nl) (TW); +31 43 388 2222

^6^ Neonatology, Department of Pediatrics, Maastricht University Medical Center +, 6202 AZ Maastricht, the Netherlands; +31 43 387 6543

^7^ Division of Obstetrics and Gynecology, The University of Western Australia, Crawley WA 6009, Australia; [alan.jobe@cchmc.org](mailto:alan.jobe@cchmc.org) (AJ), [masatoshi.saito.b4@tohoku.ac.jp](mailto:masatoshi.saito.b4@tohoku.ac.jp) (MS), [matthew.kemp@uwa.edu.au](mailto:matthew.kemp@uwa.edu.au) (MK); +61 86 488 6000

^8^ Division of Neonatology/Pulmonary Biology, The Perinatal Institute, Cincinnati Children’s Hospital Medical Center, University of Cincinnati, Cincinnati, OH 45229, USA; +1 513 636 4200

^9^ Center for Perinatal and Neonatal Medicine, Tohoku University Hospital, Sendai, Miyagi 980-8574, Japan; +81 22 717 7000

^10^ School of Veterinary and Life Sciences, Murdoch University, Perth WA 6150, Australia; +61 130 065 2494

^11^ Department of Biomedical Engineering (BMT), School for Cardiovascular Diseases (CARIM), Maastricht University, 6200 MD Maastricht, the Netherlands; +31 43 388 2222


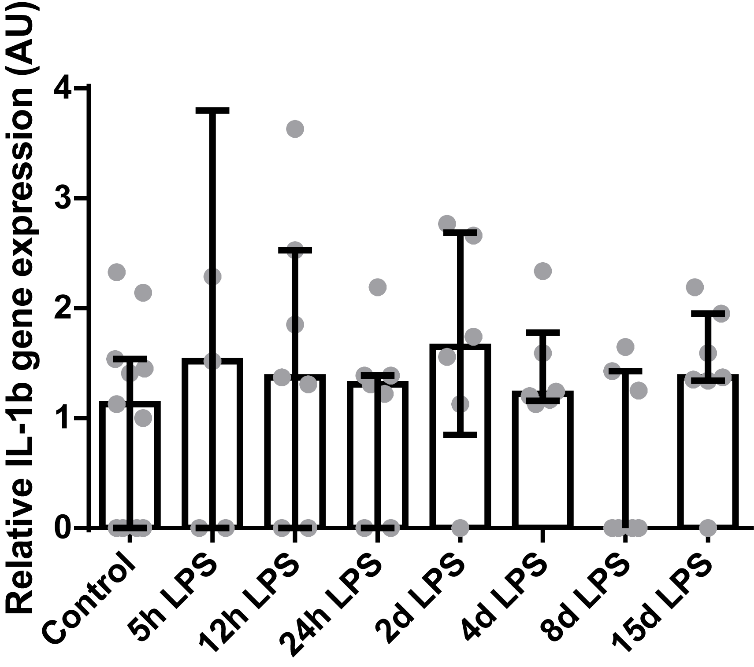


**Supplementary Figure S1.** Relative mRNA expression of *IL-1β* in AU in the liver. Hepatic *IL-1β* mRNA levels did not differ between the groups.

**
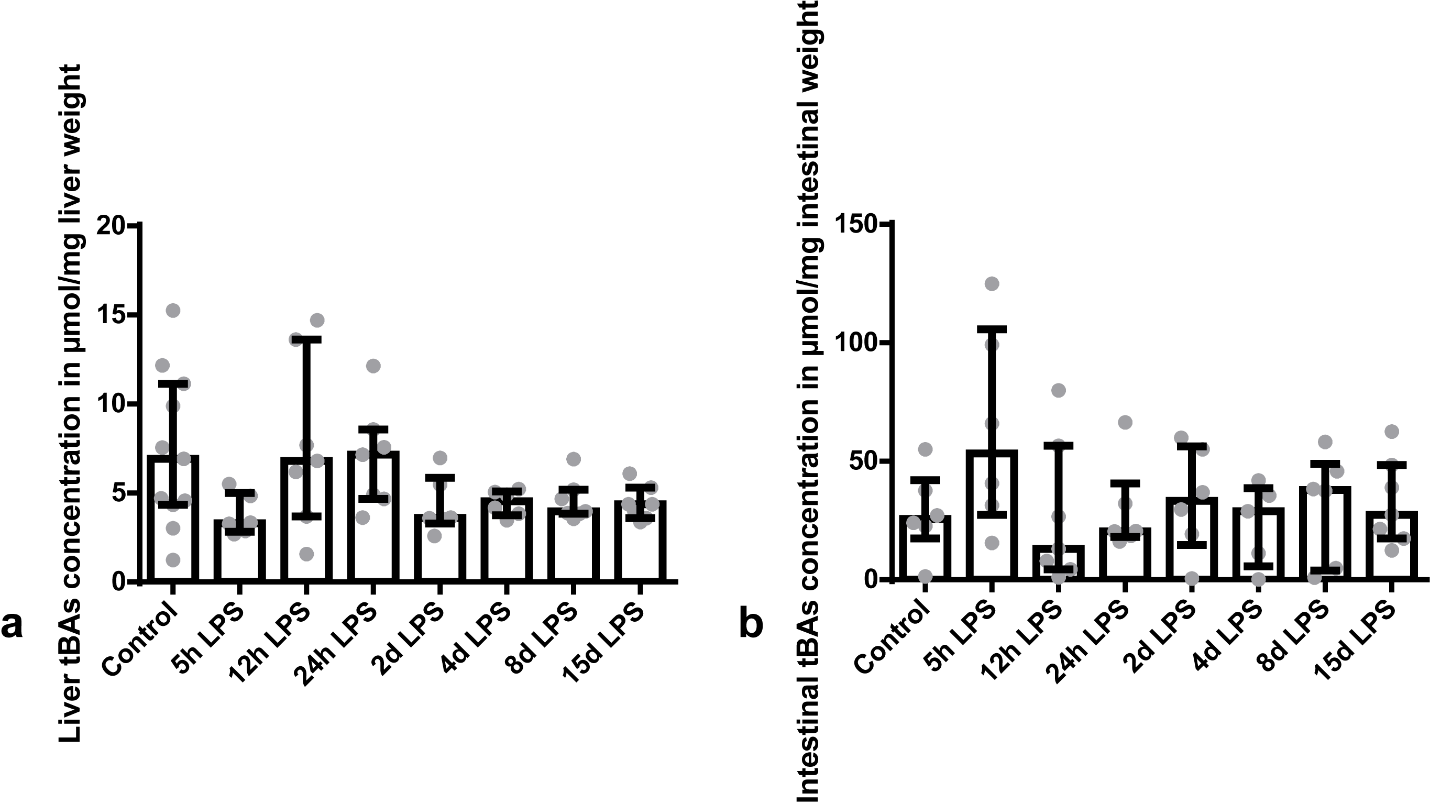
Supplementary Figure S2.** tBAs concentrations in the liver (a) and ileum (b) in μmol/L. tBAs concentrations in liver and ileum homogenates did not differ between the groups.


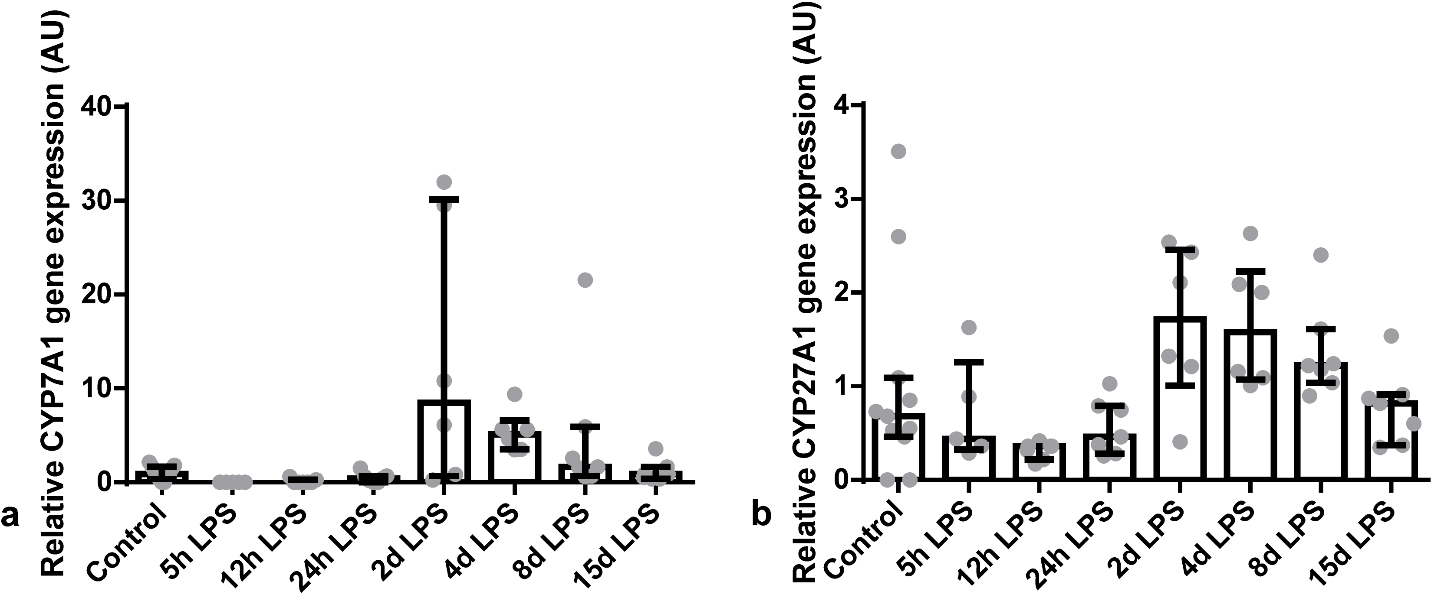


**Supplementary Figure S3.** Relative mRNA expression of *CYP7A1* (a) and *CYP27A1* (b) in AU in the liver. Gene expression levels of *CYP7A1* and *CYP27A1* did not differ among the groups.

**
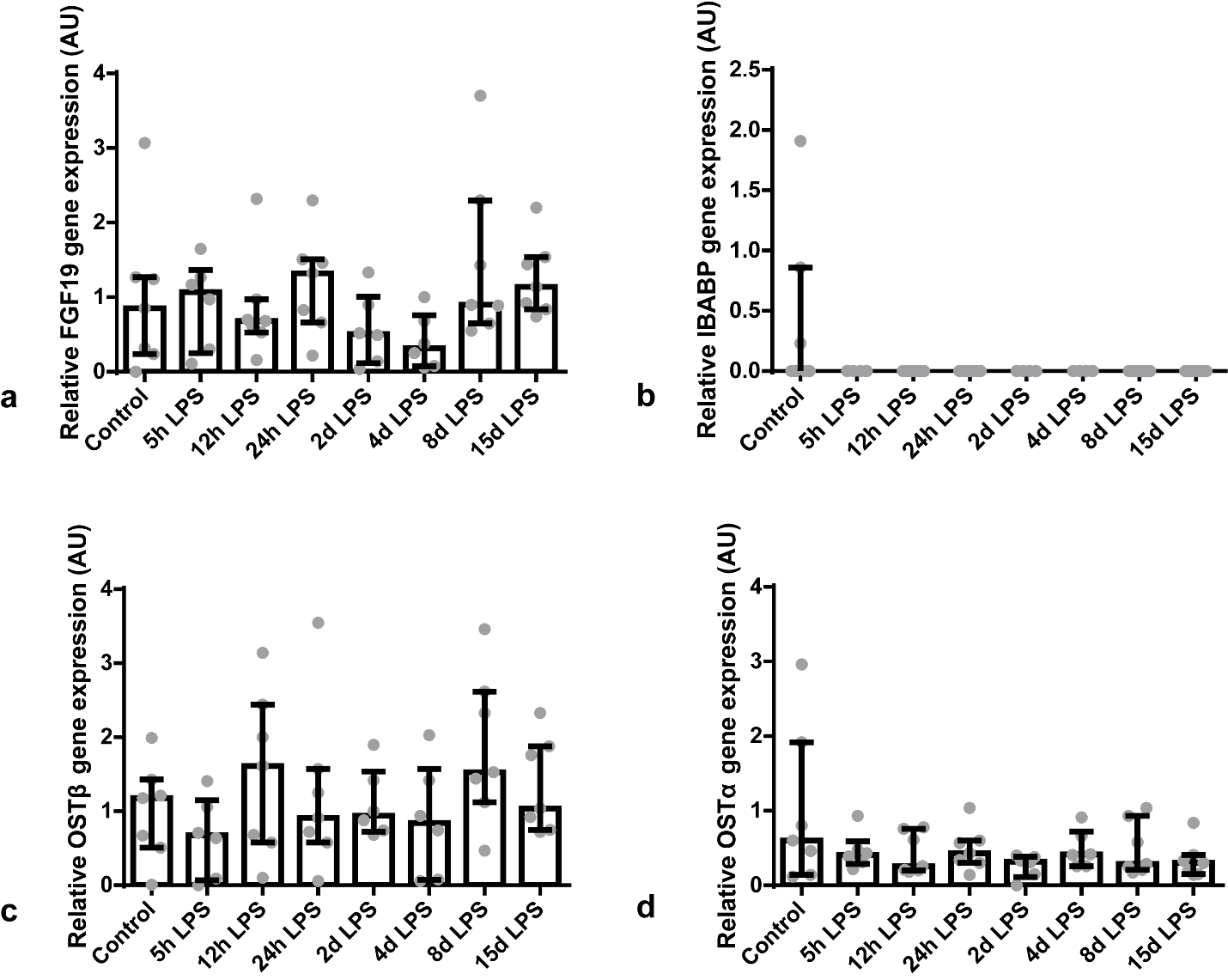
Supplementary Figure S4.** Relative mRNA expression of *FGF19* (a), *IBABP* (b), *OSTα* (c) and *OSTβ* (d) in AU in the intestine. Intestinal mRNA expression levels of *FGF19*, *IBABP* and *OSTα-β* did not differ between the groups.
